# Post-Migration Stressors and Their Association With Symptom Reduction and Non-Completion During Treatment for Traumatic Grief in Refugees

**DOI:** 10.3389/fpsyt.2020.00407

**Published:** 2020-05-27

**Authors:** A.A.A. Manik J. Djelantik, Annemiek de Heus, Diede Kuiper, Rolf J. Kleber, Paul A. Boelen, Geert E. Smid

**Affiliations:** ^1^ARQ Centrum’45, Diemen, Netherlands; ^2^ARQ National Psychotrauma Centre, Diemen, Netherlands; ^3^Department of Clinical Psychology, Faculty of Social Sciences, Utrecht University, Utrecht, Netherlands; ^4^University of Humanistic Studies, Utrecht, Netherlands

**Keywords:** traumatic loss, persistent complex bereavement disorder, posttraumatic stress disorder, day treatment program, brief eclectic psychotherapy, refugees, post-migration stressors

## Abstract

**Background:**

Resettled refugees exposed to trauma and loss are at risk to develop mental disorders such as posttraumatic stress disorder (PTSD) and persistent complex bereavement disorder (PCBD). Post-migration stressors have been linked to poor mental health and smaller treatment effects.

**Aim:**

Our aim was to evaluate reductions in PTSD and PCBD symptoms and to explore the presence of post-migration stressors and their associations with symptom change and non-completion in a traumatic grief focused treatment in a cohort of refugees.

**Methods:**

Paired sample t-tests were used to test the significance of the symptom reductions in PTSD and PCBD symptoms during treatment. The presence of post-migration stressors was derived from a qualitative analysis of the patient files. Associations between post-migration stressors and symptom reductions as well as non-completion were calculated.

**Results:**

In this uncontrolled study, 81 files of consecutive patients were included. Significant reductions in both PCBD and PTSD symptomatology with medium effect sizes were found. Patients experienced a mean of three different post-migration stressors during the treatment. Undocumented asylum seekers were more likely to be non-completers. Ongoing conflict in the country of origin was associated with smaller PTSD symptom reductions and the total number of post-migration stressors was associated with smaller PCBD symptom reductions.

**Conclusions:**

Treatment for resettled refugees for traumatic grief coincides with alleviations in both PCBD and PTSD symptomatology. Specific post-migration stressors were associated with reduced treatment effects and increased non-completion. This is a first step towards well-informed improvements of mental health interventions for resettled refugees.

## Highlights

- We evaluated reductions in PCBD and PTSD symptoms during a traumatic grief focused treatment in a treatment seeking clinical refugee sample.
- Although many post-migration stressors were present, significant symptom reductions with a medium effect sizes were observed.
- Undocumented asylum seekers were more likely to not complete the treatment.
- Ongoing conflict in the country of origin and the total number of post-migration stressors were associated with decreased symptom reductions.
- Clinicians treating mental health disorders in resettled refugees should consider the effects of post-migration stressors to prevent treatment drop-out and manage treatment expectations.

## Introduction

Resettled refugees in Western countries commonly have been exposed to traumatic and loss events due to armed conflicts, persecution and/or natural disasters in their countries of origin (1, 2). It is not surprising that in this group mental disorders such as posttraumatic stress disorder (PTSD) are frequently observed with prevalence’s varying between 35 to 47% (3–7). Recently, persistent complex bereavement disorder (PCBD), characterized by debilitating and prolonged grief, has been recognized as another form of psychopathology commonly seen in refugees seeking mental health support following loss and trauma with reported prevalence’s varying between 6 to 10% in general refugee populations and 16% to 21% in bereaved refugee populations (7–11). PCBD has been included as a condition for further study in the 5th edition of the Diagnostic and Statistical Manual of Mental Disorders (12). An equivalent syndrome named Prolonged grief disorder (PGD) was recently included in the 11th edition of the International Classification of Diseases (ICD) (13, 14). An estimated 10% of bereaved adults confronted with natural loss will develop PCBD (15). However, unexpected and violent losses and the loss of close kin (partner or child) have been associated with substantially higher rates of psychopathology than anticipated and non-violent losses and losses other than close kin (16, 17). In a recent study, it was shown that the symptom network of PCBD is closely associated with the symptom networks of PTSD and depression (9), sometimes labeled together as “traumatic grief” (18, 19). Nevertheless, treatments of mental disorders following violent loss focusing both on PTSD and PCBD at the same time are scant and have not been evaluated in refugee samples yet (18, 20). In a first study with a naturalistic design among 16 consecutive patients, a treatment program for traumatic grief for refugees was found feasible and coincided with significant declines in PTSD symptoms (18). While these results are promising, evaluation of the treatment in a larger cohort of patients, focusing on changes in both PTSD and PCBD symptoms, is needed. We will explain the treatment more thoroughly in the *Methods* section.

Refugees resettling in a new country often experience post-migration stressors and these stressors have been linked to poor mental health outcomes. These stressors include (a) socio-economic factors, i.e., financial and housing security and work problems; (b) social and interpersonal factors, i.e., family separation, family reunification, lack of social support, changes in social roles, discrimination, and changes in socioeconomic status, (c) process and immigration policies, i.e., detention, time of the asylum-seeking process, limited duration of residence permit, and as a consequence, living difficulties such as family conflict and unstable housing (21, 22).

The association between post-migration stressors and treatment outcome is an important research topic because these stressors may interfere with successful treatment conditions in several ways (23, 24). First, refugee patients may be too occupied by managing and arranging solutions for the post-migration stressors and this could lead to compromised treatment adherence. Second, in the treatment sessions there may be less time to conduct treatment, because the discussion of the current social stressors takes up too much time. Furthermore, sometimes the refugee may ask the clinician to provide assistance for their post-migration problems, which may result in ethical dilemmas for the clinician and slow down the treatment process. Lastly, when financial and housing problems are present, the costs for the transportation or treatment may be too high for the refugee, and he/she may withdraw from treatment. To the best of our knowledge, the association of these post-migration stressors with treatment outcome in resettled refugees has been only partially investigated in two studies (25, 26). Sonne et al. (25) investigated the post-migration stressors “employment status” and “integration” and found that not being employed was significantly, albeit weakly, correlated with poorer treatment outcome. Additionally, Schick et al. (26) found a correlation between a decrease of the amount of post-migration stressors “employment status”, “trauma exposure” and “visa status” over the time of treatment and better outcomes in anxiety and depression symptomatology. Importantly, an elaborate investigation of the presence of post-migration stressors and their association with drop-out rate or treatment outcome among refugees in a grief focused treatment has not yet been performed.

In sum, the purpose of this uncontrolled study was twofold. Firstly, our aim was to evaluate a traumatic grief focused treatment in a larger cohort of refugees than the previous feasibility study among 16 patients (18) and to include both the reductions in PTSD and PCBD symptoms. Because PTSD and PCBD are likely to co-occur in these patients (9) we were also interested in the correlation between PTSD and PCBD outcomes. We expected a medium size treatment effect for both PTSD and PCBD symptom reductions, based on the PTSD symptom change found in the feasibility study (18), and a correlated decline in PCBD and PTSD symptoms. Secondly, our aim was to explore the associations between postmigration stressors and treatment non-completion and examine associations between post-migration stressors and reductions in PCBD and PTSD symptomatology. We hypothesized that post-migration stressors would be associated with a higher drop-out rate and smaller symptom reductions during treatment.

## Methods

### Patients and Procedure

This is a pre and post study design. The study used data from a convenience sample of all consecutive patients who participated in the day patient treatment for traumatic grief (DPT-TG) between October 2014 and October 2018 at ARQ National Psychotrauma Center, Netherlands, which receives nationwide referrals for specialized treatment of refugees and other traumatized groups. Patients who were referred for specialized treatment of psychotrauma were considered for inclusion in the DPT-TG if it was concluded from clinical evaluation that the death of one or more loved ones was a core traumatic event and the patient endorsed clinically relevant symptoms of grief and PTSD related to this event. In addition, patients needed to be willing to engage in group treatment. These clinical evaluations were carried out by psychiatrists and clinical psychologists as well as by psychiatric residents and clinical psychologists in training under direct supervision of a psychiatrist. Exclusion criteria for participation were acute or active suicidality (i.e., current suicide plans or suicidal behaviors or suicide attempts), severe psychotic disorder, and/or severe alcohol or substance abuse. Patients underwent standardized measurements at the start (T1) and at the end (T2) of the DPT-TG. Measures were administered by a team of independent psychologists and psychiatrists, all trained in diagnostics and receiving regular supervision from senior clinical psychologists. Measurements were part of the Routine Outcome Measurements (ROM). The medical ethics committee of Leiden University was consulted, and the study was exempted from formal review because the primary purpose of ROM is not research oriented. All patients were informed during the ROM that their answers could be anonymously used for research purposes and could object if they did not agree. None of the patients eligible for inclusion in the current study expressed objections. The data were handled confidentially, and the patient identities were only available for a small group of project managers.

### Intervention

The DPT-TG is a multidisciplinary treatment program for bereaved refugees that includes exposure-based psychotherapy focused on both separation and traumatic distress. In addition, the program aims to stimulate the patients’ social activity and reinforce their social networks, and offers support in relation to legal issues, work, and education. The treatment consists of a one-year weekly 5-h program divided in three phases of each 4 months. The first phase is a stabilization phase intended to increase patients’ understanding of their symptoms and to become acquainted with the group and the treatment. The second phase focuses on processing the traumatic loss. During this phase, weekly sessions of individual brief eclectic psychotherapy for traumatic grief (BEP-TG) (19) are offered. BEP-TG consists of 16 sessions and was carried out by trained therapists (i.e. psychologists and psychiatrists). The third phase is focused on resocialization. Concrete individual future orientated goals are addressed in the group therapy and patients are encouraged to strengthen their social networks and assisted in applying for jobs or voluntary work (18). Patients underwent standardized measurements in the first four months of the DPT-TG, but before the start of the BEP-TG (T1), and in the last 4 months of DPT-TG, when the BEP-TG was finished (T2). DPT-TG have been found feasible in a prior study of 16 patients (18). More information about the schedule can be found in **Supplementary Materials A**. On average, patients attended a mean of 74% of the psychotherapy sessions (SD *=* 14.08, range 35%–100%).

### Measurements and Data Collection

#### Traumatic Grief Inventory-Self Report (TGI-SR)

To assess the intensity of grief symptoms, the TGI-SR (27) was administered. The TGI-SR consists of two parts. The first part measures lifetime losses of loved ones and the second part assesses the intensity of the grief symptoms as experienced by the client in the past month. In case of multiple losses, the loss that is currently considered most painful is used as the anchor event for the second part. This consists of 18 items to assess the intensity of grief reactions rated on a 5-point Likert scale, ranging from “1 = never” to “5 = always”, e.g., “I had intrusive thoughts and images associated with his/her death”; “I felt a strong longing or yearning for the deceased.” A questionnaire diagnosis of probable PCBD according to DSM-5 was assigned to patients who scored 4 (“often”) or 5 (“always”) on at least one core symptom, at least six out of 12 additional symptoms, and the dysfunction criterion. The TGI sum score provides an index of severity of the grief symptoms, with a higher score associated with higher grief severity (range, 18 to 90). The TGI-SR has been validated in both a patient and a community sample (28). The internal consistency in the current study was good (Cronbach’s alpha at T1 =.84 and at T2 =.92).

#### Clinician-Administered PTSD Scale 5 (CAPS-5)

PTSD symptom severity was assessed with the CAPS-5 (29). The CAPS-5 is a 30-item clinician rated interview, comprising 20 items assessing PTSD symptoms according to the DSM-5 (12). The intensity and frequency of symptoms during the past month are separately rated and then combined to form a single severity score for each item. Severity scores are rated on a 5-point scale ranging from 0 (absent) to 4 (extreme/incapacitating), resulting in a total score range of 0 to 80. A diagnosis of PTSD according to DSM-5 was assigned to patients who scored ≥ 2 on at least one symptom of criterion B and C, at least two symptoms of criterion D and E, and criterion F (disturbance has lasted 1 month) and G (dysfunction criterion). An initial evaluation showed good psychometric properties for the CAPS-5 (29). The internal consistency in the current study was good (Cronbach’s alpha at T1 =.86 and at T2 =.91). For one participant PTSD symptoms were assessed by the Clinician Administered PTSD Scale for DSM-IV [CAPS-4 (30)]. To be able to compare the scores of both CAPS versions, the sum scores were converted into probability scores, i.e., on a scale from 0 (lowest possible score) to 1 (highest possible score).

#### Socio-Demographic Information and Post-Migration Stressors

Socio-demographic information and the presence of post-migration stressors in the current patient sample were examined using information retrieved from patient files. Each file, including the admission report and notes from the therapists, was examined for the presence of post-migration stressors by AH and DK. The first three authors created a list of post-migration stressors using information from the review by Li, Liddell (21). Subsequently, additions and removals of this list of post-migration stressors were made by exploring similarities and differences across the files by AH and DK. When in doubt, the findings were discussed with the first author AD until consensus was reached. For the stressor “ongoing conflict in the country of origin”, we searched the internet to find evidence of conflict at the time of treatment (**Supplementary Materials B**).

### Patient Characteristics

Of the 81 patients who started, 57 patients completed the full year (completers) and 24 patients did not complete DPT-TG (non-completers). Fourteen patients dropped out completely from DPT-TG before the BEP-TG module was finished. Ten patients were not able to attend the weekly one-day treatment sessions, but continued treatment at the outpatient clinic with a customized program due to pregnancy and/or psychosocial problems. Eight patients did not complete at least one of the questionnaires due to various reasons such as psychosocial problems or illnesses in the week of the administration of the questionnaires but completed the rest of the DPT-TG. We included a flow chart in **Supplementary Materials C** with pre- and post-questionnaire numbers and reasons for drop-out. On average, patients were 42 years old (SD = 9.24). Most patients were male (84%) and originated from the Middle East (58%). Patients who had lost a child or partner were slightly more likely to drop-out or receive a customized treatment (**Table 1**).

**Table 1:** Pretreatment socio-demographic and loss-related characteristics and symptom levels of the completers and early drop-outs or customized treatment in a refugee sample.

	Completers of DPT-TG (*n* = 57)	Early drop-outs or customized treatment (*n* = 24)	Significance test for differences between groups	*p* Value
Age, M (SD)	42 (9.43)	42 (9.57)	t(79) = 0.13	.84
Gender, *n* (%)			χ^2^(1, *n* = 80) *=* 0.03	.86 Fisher’s exact test: 1.00
Female	9 (16)	4 (17)		
Male	48 (84)	19 (83)		
Marital status, *n* (%)			χ^2^(3, *n* = 81) = 1.89	.56 Fisher’s exact test: .62
Single	14 (25)	7 (29)		
Married	32 (56)	12 (50)		
Divorced	8 (14)	2 (8)		
Widowed	3 (5)	3 (13)		
Level of education, *n* (%)			χ^2^(2, *n* = 75) = 1.88	.39 Fisher’s exact test: .37
Low	10 (18)	6 (25)		
Middle	35 (61)	10 (42)		
High	9 (16)	5 (21)		
Region of origin, *n* (%)			χ^2^(4, *n* = 81) = 2.96	.57 Fisher’s exact test: .50
Dutch/Colony	2 (5)	3 (13)		
Middle East	34 (60)	12 (50)		
Africa	11 (19)	4 (16)		
Bosnia Herzegovina/Serbia	9 (16)	4 (17)		
Asia	1 (2)	1 (4)		
Missing Family, *n* (%)	10 (17.5)	4 (17)	χ^2^(1, *n* = 81) = 0.01	.92 Fisher’s exact test: 1.00
Number of losses, M (SD)	5.59 (2.14)	5.65 (2.46)	t(75) = 0.11	.92 Fisher’s exact test: 1.00
Relationship to lost loved one^1^, *n* (%)			χ^2^(5, *n* = 60) = 10.81	.055 Fisher’s exact test: .038
Partner	1 (1.8)	3 (12.5)		
Child	4 (7.0)	2 (8.3)		
Parent(s)	15 (26.3)	5 (20.8)		
Sibling	8 (14.0)	3 (12.5)		
Friend	15 (26.3)	1 (4.2)		
Other	1 (1.8)	2 (8.3)		
Violent loss a, *n* (%)	47 (83)	20 (83.3)	χ^2^(1, *n* = 78) = 0.03	.86 Fisher’s exact test: 1.00
PCBD according to TGI-SR, *n* (%)	30 (53)	13 (54)	χ^2^(1, *n* = 81) = 0.02	.90 Fisher’s exact test: 1.00
PTSD according to CAPS, *n* (%)	51 (90)	21 (88)	χ^2^(1, *n* = 78) = 0.74	.39 Fisher’s exact test: .67
Number of traumatic events, M (SD)	29 (5.24)	19 (4.48)	t(64) = 0.75	.87 Fisher’s exact test: .76
Psychiatric medication, *n* (%)	41 (72)	15 (63)	χ^2^(1, *n* = 72) = 0.12	.73 Fisher’s exact test:.48
Psychiatric comorbidity at during clinical intake assessment, *n* (%)				
Depressive disorder	45 (79)	20 (83)	χ^2^(1, *n* = 81) = 0.21	.65 Fisher’s exact test: .77
Substance abuse	8 (14)	6 (25)	χ^2^(1, *n* = 81) = 1.42	.23 Fisher’s exact test: .33
Dissociative disorder	2 (4)	0 (0)	χ^2^(1, *n* = 81) = 0.86	.35 Fisher’s exact test: 1.00
Disruptive, Impulse-Control, and Conduct Disorders	2 (4)	0 (0)	χ^2^((1, *n* = 81) = 0.86	.35 Fisher’s exact test: 1.00
Somatic Symptom and Related Disorders	3 (5)	0 (0)	χ^2^(1, *n* = 81) = 1.31	.25 Fisher’s exact test: .56
Schizophrenia and other psychotic disorders	1 (2)	1 (4)	χ^2^((1, *n* = 81) = 0.41	.52 Fisher’s exact test: .51
Sexual Disorders	1 (2)	0 (0)	χ^2^(1, *n* = 81) = 0.43	.51 Fisher’s exact test: 1.00
Obsessive Compulsive Disorders	2 (4)	0 (0)	χ^2^(1, *n* = 81) = 0.86	.35 Fisher’s exact test: 1.00
Eating Disorder	1 (2)	0 (0)	χ^2^(1, *n* = 81) = 0.43	.51 Fisher’s exact test: 1.00
Indications for probable personality disorder	32 (56)	13 (54)	χ^2^(1, *n* = 81) = 0.03	.87 Fisher’s exact test: 1.00

^1^Concerning the loss that is considered most painful; PCBD, persistent complex bereavement disorder; PTSD, Posttraumatic Stress Disorder; DPT-TG, Day Patient Treatment for Traumatic Grief.

### Statistical Analysis

#### Missing Data

Missing data at item level were considered missing at random. Two items on the TGI-SR were missing for one participant at T2. For this participant, we calculated the total TGI-SR score using the mean of the valid items multiplied by the total number of items.

The missing data regarding social stressors and sum scores of PCBD and PTSD were handled using listwise deletion. The significance threshold was set at *p* < .05 for all analyses.

#### Power Calculation

First, we calculated the effect size from the information of the first 16 patients of the study of de Heus, Hengst (18) together with 13 consecutive patients (in total 29 patients) with the program Gpower (31). Based on this sample, we expected to find a difference between the pre and post measurements in the current study with a small to medium effect size (PTSD: d = 0.5, PCBD: d = 0.4, power=.80; α=.05). For PTSD, a sample size of 34 completers was needed to calculate the effect size with t-test and a sample size of 63 completers was needed to calculate the effect size with analysis of variance (ANOVA). For PCBD, a sample size of 52 completers was needed to calculate an effect size with t-test and a sample size of 69 completers was needed to calculate the effect size with ANOVA. Because, we only had 57 patients who completed the DPT-TG, we used the t-test for the effect size calculations in the following sections.

#### Aim A: PCBD and PTSD Symptom Reductions During Treatment

We used the data of all patients who completed DPT-TG as well as the TGI-SR or CAPS on both timepoints. We performed a paired samples t-test to test the statistical significance of symptom reduction (32). Diagnostic status with regard to traumatic grief was operationalized as a categorical outcome variable that could take on the following categories: PCBD and PTSD, PTSD only, PCBD only, or no PTSD or PCBD. We used the McNemar test to test the statistical significance of the change within each of the diagnostic categories (33). To assess whether a change in diagnostic category membership had occurred within the sample as a whole across the two time points, we applied the marginal homogeneity test (33). Additionally, we calculated the Cohen’s d to assess the effect size. We performed this with the TGI-SR sum score and the two subscales symptom clusters of PCBD, namely separation distress and reactive distress and social/identity disruption. We repeated this with the CAPS sum score and PTSD subscales, namely intrusive symptoms, avoidance of stimuli associated with the event, negative changes in cognitions and mood, and marked alterations in arousal and reactivity. We interpreted the effect sizes as small (d = 0.2), medium (d = 0.5), and large (d = 0.8) (34). Lastly, we calculated the correlation between PCBD and PTSD symptom reductions. We used the expectation-maximization (EM) algorithm as an analysis of the sensitivity of the correlation estimate to the incompleteness of our data as described in Dempster, Laird (35). This algorithm accounts for the missing data using maximum-likelihood estimates (36). For all analyses, we used SPSS version (37), except for the McNemar test which we performed in R with package “exact2x2”.

#### Aim B: Post-Migration Stressors and the Association With Treatment Drop-Out and Symptom Reductions During Treatment

We determined whether or not the group of DPT-TG completers (both the patients with complete and missing questionnaires; *n* = 57) and DPT-TG non-completers (*n* = 24) differed significantly in terms of post-migration stressors, using t-tests, chi square and Fisher exact tests. We used both chi square and Fisher exact tests because, due to our sample size, some of the expected frequencies of the post-migration stressors were smaller than 5 (38). In addition, we evaluated the associations between post-migration stressors and symptom reductions. To account for both the initial differences between scores at T1 and for measurement error inherent in the use of repeated measures on the same instrument, we first calculated the residual gain score for PCBD and PTSD (39). We regressed the sum scores at T2 on the sum scores at T1 and saved the residuals to use these as residual gain scores. Then, we examined whether these residual gain scores varied as function of the post-migration stressors using one-way ANOVA. For all analyses, we used SPSS version (37), except for the Fisher exact test which we performed in R with package “exact2x2”.

## Results

### Aim A: PTSD and PCBD Symptom Reduction During Treatment

**Table 2** shows an overview of the reductions in PCBD and PTSD symptomatology during DPT-TG. As shown in **Table 2**, patients scored significantly lower on the post-treatment grief measurement compared to the pre-treatment grief measurement [pre intervention: M = 66.80 (SD = 11.01), post intervention: M = 58.92 (SD = 14.71), p < .001]. With respect to the effect size we found a medium effect for PCBD and PTSD (*d* =.61 and *d* =.33, respectively) (35). PCBD diagnoses decreased from 54% (29 patients) at T1 to 41% (22 patients) at T2. PTSD diagnoses dropped from 89% (46 patients) at T1 to 70% (36 patients) at T2 (**Table 2**). Within each of the diagnostic categories (PCBD and PTSD, PTSD only, PCBD only, or no PTSD or PCBD), there was no significant change over time. For the sample as a whole, we found a significant change in diagnostic category membership across the two assessment times: standardized MH statistic = 3.11, p =.002.

**Table 2:** Symptom reductions and diagnostic changes in the Day Patient Treatment for Traumatic Grief Completers.

	Pre-treatment score M (SD)	Post treatment score M (SD)	Significance test for differences between groups	*p* Value	Cohen’s d
*Symptom reductions PCBD* *Patients who filled in both PCBD questionnaires (n = 54)*					
PCBD sum score, M (SD)	66.80 (11.01)	58.92 (14.71)	t(54) = 3.91	<.001	0.61
subdomain: separation distress. M (SD)	4.17 (0.49)	3.69 (0.89)	t(54) = 4.72	<.001	0.70
subdomain: reactive distress and social/identity disruption. M (SD)	3.56 (0.72)	3.14 (0.85)	t(54) = 3.33	.002	0.53
subdomain: functional impairment. M (SD)	3.68 (1.29)	3.19 (1.30)	t(54) = 2.63	.005	0.38
Patients endorsing separation distress, *n* (%)	53 (98.2)	44 (81.5)		.42	
Patients endorsing reactive distress and social/identity disruption, *n* (%)	49 (90.7)	47 (87.0)		.92	
Patients endorsing functional impairment, *n* (%)	34 (63.0)	27 (50.0)		.44	
*Symptom reductions PTSD* *Patients who completed both PTSD interviews (n = 52*)					
PTSD sum score, M (SD)	0.54 (0.15)	0.48 (0.19)	t(52) = 2.92	.005	0.34
Criterium B: intrusive symptoms M (SD)	0.64 (0.19)	0.54 (0.18)	t(52) = 3.44	<.001	0.55
Criterium C: avoidance of reminders of the event M (SD)	0.55 (.215)	0.41 (0.26)	t(52) = 3.49	<.001	0.62
Criterium D: negative alterations in cognitions and mood M (SD)	0.52 (0.20)	0.49 (0.24)	t(52) = 1.17	.25	0.15
Criterium E: alterations in arousal and reactivity M (SD)	0.47 (0.16)	0.44 (0.17)	t(52) = 1.10	.28	0.15
Patients meeting criterium B, *n* (%)	51 (98.1)	47 (90.4)		.92	
Patients meeting criterium C, *n* (%)	52 (100)	44 (84.6)		.48	
Patients meeting criterium D, *n* (%)	48 (92.3)	42 (80.8)		.60	
Patients meeting criterium E, *n* (%)	49 (94.2)	46 (88.5)		.84	
*Change in diagnostic status* *Participants who filled in both PCBD and PTSD questionnaires at pre-treatment (N=56) and post-treatment (N=53)*					
Patients endorsing the PTSD diagnosis	46 (89)	36 (70)		.32	
Patients endorsing the PCBD diagnosis	29 (54)	22 (41)		.40	
PCBD and PTSD N, (%)	27 (48)	17 (32)		.17	
PTSD only N, (%)	23 (41)	20 (38)		.76	
PCBD only N, (%)	2 (4)	6 (11)		.29	
No PTSD and PCBD	4 (7)	10 (19)		.18	
Change in diagnostic category membership within sample as a whole				.002	

DPT-TG, day patient treatment–traumatic grief; PCBD, Persistent complex bereavement disorder; PTSD, Posttraumatic stress disorder.

The symptom reduction of PCBD and PTSD was highly correlated [*r*(49) =.60, p < .001)], also when applying expectation-maximization to account for the missing questionnaires [*r*(57) =.59].

### Aim B: Post-Migration Stressors and the Association With Treatment Drop-Out and Symptom Reductions During Treatment

The following list of post-migration stressors were identified: time in Netherlands, duration of the asylum period, refugee status, language problems, work situation, housing problems, family separation of close kin, ongoing conflict in country of origin and total number of post-migration stressors. The categories of the post-migration stressors are specified in **Table 3**. Three patients experienced a change in legal status during treatment. One participant received a temporary permit, one participant lost a temporary permit and became undocumented, and one participant received, lost and received again a temporary permit during the year of treatment. Although these are interesting findings, the sample size was too small to be included in the further analyses.

**Table 3:** Post-migration stressors pre- or during treatment in the completers and early drop-outs or customized treatment group of the refugee sample.

	Completers of DPT-TG (*n* = 57)	Early drop-outs or customized treatment (*n* = 24)	Significance test for differences between the groups	p Value
Time in the Netherlands (months), M (SD)	191.40 (104.85)	192.40 (107.56)	t(73) = 0.29	.97
Time asylum period (months), M (SD)	66.74 (59.41)	87.40 (102.74)	t(35) = 5.99	.45
Legal status, *n* (%)			χ^2^(3, *n* = 81) = 10.0	.002 Fisher’s exact test: .022
Permanent permit	46 (81)	16 (67)		
Temporary permit	7 (12)	3 (13)		
Pending	3 (5)	0 (0)		
Undocumented	1 (2)	5 (21)		
Language, *n* (%)			χ^2^(1, *n* = 81) = 0.01	.92 Fisher’s exact test: 1.00
Dutch	41 (72)	17 (71)		
Insufficient Dutch proficiency	16 (28)	7 (29)		
Work situation, *n* (%)			χ^2^(3, *n* = 81) = 2.93	.40 Fisher’s exact test: .43
Employed	2 (4)	3 (13)		
Sick leave	10 (18)	3 (13)		
Disabled	8 (14)	2 (8)		
Unemployed	37 (65)	16 (67)		
Housing problems, *n* (%)	12 (21)	8 (33)	χ^2^(1, *n* = 81) = 1.37	.24 Fisher’s exact test: .27
Family separation close kin, *n* (%)	18 (32)	9 (38)	χ^2^(1. *n* = 81) = 0.27	.70 Fisher’s exact test: .62
Ongoing conflict in country of origin, *n* (%)	30 (53)	8 (33)	χ^2^(1. *n* = 81) = 2.53	.11 Fisher’s exact test: .15
Total number of stressors M (SD)	2.86 (1.76)	2.83 (1.81)	t(79) =.72	

DPT-TG, Day patient treatment–traumatic grief.

On average, patients experienced three different post-migration stressors during the treatment. Undocumented asylum seekers were more likely to be non-completers (**Table 3**). Some of them completely dropped out from DPT-TG (*n* = 3) and others received a customized treatment (*n* = 2). Other post-migration stressors were not significantly associated with treatment non-completion. The post-migration stressor “ongoing conflict” was inversely associated with PTSD symptom reductions. Total number of different post-migration stressors was inversely associated with PCBD symptom reductions (**Table 4**).

**Table 4:** The associations between the post-migration stressors pre- and during treatment and the TGI-SR and CAPS residual gain score.

	TGI-SR RES				CAPS RES			
	F	df1	df2	*p* value	F	df1	df2	*p* value
Legal status	1.07	3	50	.37	0.41	3	48	.75
Language	1.17	1	52	.29	0.01	1	50	.94
Work situation	0.90	3	50	.45	1.78	1	50	.19^a^
Housing problems	1.21	1	52	.28	0.14	1	50	.72
Family separation close kin	0.68	1	52	.41	1.01	1	50	.41^b^
Ongoing conflict in country of origin	1.36	1	52	.25^b^	4.83	1	50	.033^b^
Total number of stressors	4.67	1	52	.04	0.54	1	50	.47

a Due to the violation of the assumptions regarding outliers, normality and homogeneity, we have dichotomized “work situation” for this analysis into employed versus not employed. We also performed a robust regression of the original (non-dichotomized) “work situation” variable using the package “MASS” in R. For the TGI-SR difference the t-value was 0.51, for the CAPS difference the t-value was 0.97; both values were not significant.

b Due to the violation of the assumption regarding homogeneity we have reported the Welsh test.

CAPS RES, Clinician Administered Posttraumatic Stress Disorder residual score; TGI-SR RES, Traumatic Grief Inventory Residual score.

## Discussion

In this study on symptom reductions during treatment of traumatic grief in resettled refugees, a significantly correlated reduction of both PCBD and PTSD symptomatology was found with a medium effect size. Post-migration stressors were associated with both poor treatment completion and smaller symptom reductions. More specifically, not having a legal permit was associated with poor treatment completion. Ongoing conflict in the country of origin and a higher total number of post-migration stressors were associated with smaller reductions in PTSD and PCBD symptoms, respectively.

Our results showed that both PCBD and PTSD symptomatology can decrease simultaneously during treatment which is in line with the evidence available on the impact of grief focused treatment in other populations (20). However, more studies on the effectiveness of grief focused treatment in refugee populations are needed to verify our findings.

In our study, asylum seekers who were staying undocumented in Netherlands were more likely to drop out of the treatment fully or to require customized treatment. The evidence for the association between residency status and mental health has been contradictory. Although several studies have indicated that residence status is unrelated to the prevalence of mental disorders (5, 21, 40–42), recently, in the case of PCBD, one study found that having a resident status was associated with less symptoms (7). Undocumented asylum seekers have received a negative status decision, and have only limited access to shelter, food and health care (43). Due to the combination of post-migration stressors, legal situation and uncertain future perspective, these people may be in need for special attention to prevent drop-out. Conversely, one could imagine that an improvement in legal status might be a protective factor. However, in the current study, we were unable to examine this because there was only one patient who received a temporary permit during treatment. Future studies are needed to further elucidate the association between legal status and treatment effect. Nevertheless, all other post-migration stressors were not found to be associated with treatment drop-out. It could be that clinicians’ hesitance to start treatment with a refugee because of the fear for drop-out appears therefore unnecessary in most circumstances. However, because of our small sample, conclusive negative conclusions must remain tentative, pending further research. In our sample, ongoing conflict in the country of origin was significantly associated with less reductions in PTSD symptomatology. Nickerson et al. (44) evaluated the influence of fear for family remaining in the country of origin with an ongoing conflict and the mental health of Iraqi refugees in a cross-sectional survey. They found higher PTSD symptomatology and depression as well as greater mental health-related disability. It can be argued that mass media and contact with people in the home country about the ongoing conflict continues to trigger traumatic experiences and memories. Furthermore, worries about family members still in the conflict area exposed to armed conflicts and violence could play a role as to why treatment may not be effective for these individuals. As expected, the total number of post-migration stressors was associated with smaller treatment effects. This is in line with our clinical observations of patients who sometimes appear so occupied by managing and arranging solutions for their post-migration stressors that they are not capable to adhere to the steps of a treatment protocol. Clinicians need to provide emotional and information support for patients reporting post migration stressors, knowing that this might not have short term positive effects in mental health outcomes.

Limitations of this study must be noted. The findings of this study need to be interpreted in the light of its naturalistic (real-world) setting. It cannot be stated with certainty that the decreases in PCBD- and PTSD symptom severity can be attributed to the effects of the DPT-TG. Furthermore, we could only assess the importance of the post-migration stressors that clinicians noted in the patient files. On the one hand, one could argue that we have captured all factors that may influence the treatment process according to the clinicians and patients themselves. On the other hand, “ongoing conflict in the country of origin” was one of the few post-migration stressors found to be associated with treatment outcome and this stressor was not systematically asked by clinicians in the patient files. Therefore, there might be a lack of knowledge by clinicians about which post-migration stressors are worth assessing at the beginning of treatment and theoretically, some participants may have experienced difficulties but did not mention them in therapy. As post-migration stressors were identified at some point during the treatment and recorded in the patient files, we could not specifically determine at what point in time during the course of treatment these stressors had the largest impact. Future studies are needed to investigate the role of post-migration stressors more elaborately and standardized, by including living difficulties questionnaires in routine measures. Furthermore, our study had an uncontrolled study design and a relative small sample size, future studies need to assess the treatment effect of the DPT-TG with a control group and follow-up measurement.

Strengths of this study must also be noted. The findings of this study generate several well-informed answers to some urgent clinical questions that rise in treating resettled refugee patient populations characterized by multiple post-migration stressors. Our findings indicate that treatment for traumatic grief coincides with alleviations in both PCBD and PTSD symptoms in refugees who have faced multiple traumatic events and traumatic losses. Findings also indicate that traumatized refugees may benefit from treatment even in the presence of multiple post-migration stressors.

Implications of this study include that clinicians need to keep in mind that post-migration stressors may interfere with a smooth treatment process and that these stressors are worth documenting systematically at intake. In the case of ongoing conflict or a high number of stressors, symptom reductions may be modest, and clinicians may educate patients about these effects to manage treatment expectations and to prevent demoralization. Furthermore, special attention is needed for undocumented patients who were denied a refugee status in order to prevent them from early drop-out of treatment. Further studies are needed to investigate how to raise refugee wellbeing and assist with their resettlement in a host country can be beneficial to enhance treatment outcome in psychological interventions.

## Data Availability Statement

The ROM data of ARQ National Psychotrauma center was accessible for the authors during the time of study. The data is not publicly available. Requests to access the data should be directed to the corresponding author.

## Ethics Statement

Ethical approval was not provided for this study on human participants because Measurements were part of the Routine Outcome Measurements (ROM). We consulted the medical ethics committee of Leiden University, and the study was exempted from formal review because the primary purpose of ROM is not research oriented. All patients were informed during the ROM that their answers could be anonymously used for research purposes and could object if they did not agree. None of the patients eligible for inclusion in the current study expressed objections. Written informed consent for participation was not required for this study in accordance with the national legislation and the institutional requirements.

## Author Contributions

AD, GS, and PB were responsible for the design of the study. AD, GS, AH, and DK were responsible for the data-collection. AD was responsible for the data-analysis and AD, GS, and PB for the interpretation of the data. AH and DK contributed to the data-analysis and paragraphs of the manuscript. PB, RK, and GS supervised AD. AD wrote the drafts of the manuscript. All authors were involved in revising the draft versions critically and all authors approved the final version of the manuscript.

## Conflict of Interest

The authors declare that the research was conducted in the absence of any commercial or financial relationships that could be construed as a potential conflict of interest.
