# Supplementary material for: Post-Migration Stressors and Their Association With Symptom Reduction and Non-Completion During Treatment for Traumatic Grief in Refugees
Source: Front Psychiatry. 2020 May 27;11:407. doi: 10.3389/fpsyt.2020.00407 (PMC7273964; doi:10.3389/fpsyt.2020.00407)
Supplement: Supplementary file 1 [file Table_1.docx]

**Supplementary Materials**

**Supplementary Materials A**

Table.

Time schedule of the day patient treatment for traumatic grief (DPT-TG).

| *Time schedule* | *Phase 1* | *Phase 2* | *Phase 3* |
| --- | --- | --- | --- |
| *10.00-10.15* | *Day opening* | | |
| *10.15-11.30* | *Psycho education* | *Art therapy* | *Psycho motor therapy* |
| *11.30-11.45* | *Coffee and tea* | | |
| *11.34-13.00* | *Art therapy* | *Psychotherapy BEP-TG* | *Social orientation* |
| *13.00-13.30* | *Lunch* | | |
| *13.30-14.30* | *Psycho motor therapy* | *Socio therapy* | *Clinical and social check ups* |
| *14.30-15.30* | *Clinical and social check ups* | *Clinical and social check ups* | *Socio therapy* |

*Note*. BEP-TG = brief eclectic psychotherapy for traumatic grief.

**Supplementary Materials B**

Table.

*Conceptualization of the post-migration stressors.*

| Social stressors | Conceptualization |
| --- | --- |
| Language | In need of an interpreter vs. not in need of an interpreter. |
| Legal status | Differentiation between having a permanent permit, a temporary permit (5 years or 1 year), pending (waiting for the verdict about the status), or undocumented (having  the permit request denied) |
| Time in NL | Months since arrival in The Netherlands until start of the treatment. |
| Time of asylum period | Duration in months between arrival in The Netherlands and receiving a temporal status. |
| Housing problems | Not having housing problems versus having housing problems (defined as living on the streets, in an asylum center and/or a high dissatisfaction with current housing situation). |
| Work | Being employed, on sick leave, disabled, or unemployed at the start of treatment. |
| Family separation | Not being separated from family vs. being separated from partner and/or children, or when there is/are no partner/children, being separated from parents and/or  siblings |
| Ongoing conflict | The presence of a current ongoing conflict. (currently  is during treatment period). Information retrieved from:  https://www.crisisgroup.org/, https://www.cfr.org/, http://isdp.eu/, http://www.bbc.com/,  https://www.nederlandwereldwijd.nl/reizen/reisadviezen, https://www.state.gov/,  https://www.britannica.com, https://diplomatie.belgium.be/nl |
| Living situation | Living alone, living with partner and/or children, living separated from partner and/or children. |
| Change in legal status | Having a change in the legal status (during the treatment period). This can be the change from pending to a temporary permit or the loss of a temporary permit |

**Supplementary Materials C**

*
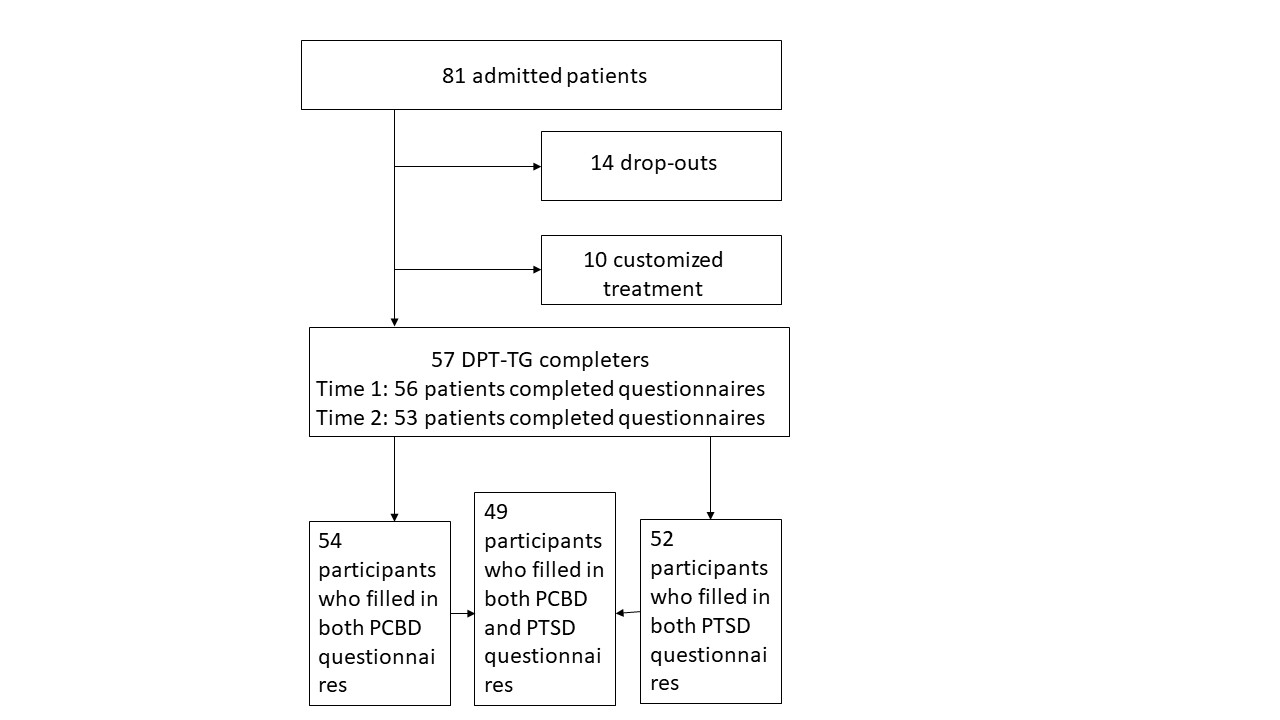
*

*Figure.* Flow chart day patient treatment for traumatic grief (DPT-TG). PCBD = Persistent complex bereavement disorder. PTSD = Posttraumatic Stress Disorder. DPT-TG = day patient treatment for traumatic grief.
